# Approach to Evaluating Accounting Informatization Based on Entropy in Intuitionistic Fuzzy Environment

**DOI:** 10.3390/e20060476

**Published:** 2018-06-15

**Authors:** Ming Li, Wendan Wei, Jialin Wang, Xiaoyu Qi

**Affiliations:** School of Business Administration, China University of Petroleum-Beijing, Beijing 102249, China

**Keywords:** accounting informatization level, evaluation index system of accounting informatization, intuitionistic fuzzy sets, cross entropy

## Abstract

Accounting informatization is an important part of enterprise informatization. It affects the accounting and finance operational efficiency. With the comprehensive evaluation of accounting informatization from multiple aspects, we can find the strengths and weaknesses of corporate accounting informatization, which then can be improved precisely. In this paper, an evaluation approach of accounting informatization is proposed. Firstly, the evaluation index system is constructed from the aspects of strategic position, infrastructure construction, implementation of accounting informatization, informatization guarantee, and application efficiency. Considering the complexity and ambiguity of the index, experts are required to give linguistic ratings which are then converted into intuitionistic fuzzy number. Then, an entropy and cross entropy method based on intuitionistic fuzzy sets is proposed to derive the weights of experts so as to reduce the error caused by personal bias. By combining the weights of the index and weighted ratings, the evaluation results are obtained. Finally, a case of accounting information evaluation is given to illustrate the feasibility of the proposed approach.

## 1. Introduction

With the promotion of accounting informatization in the construction of enterprise informatization, the importance of the implementation and application of accounting informatization is increasing. The research on accounting informatization evaluation is more and more based in academia [1,2,3] and the related research started with information systems. On the basis of the traditional theory, it is proposed to establish an accounting information system with the “event law” as the accounting theory [4]. In 1970s, information technology began to be widely applied in financial management [5]. In the late 1970s, Nolan, an expert in management information systems, put forward the six-stage theory of information systems evolution [6]. After the 1980s, the REA (Resources-Events-Agents) model that caused economic changes in the corporate value chain emerged and, based on this model, the accounting information system was designed [7]. Since the 21st century, the focus of research on accounting informatization has shifted to practical construction. Scholars have studied how to develop knowledge management systems [8]; how to apply advanced information technology tools such as expert systems and artificial intelligence, data warehouse and data mining, and CASE (Computer Aided Software Engineering) development tools for management and decision information systems [9]; and how to integrate the application of information technology with organization, strategy, management, and business process control systems.

During the implementation of information technology related to accounting, the level of accounting informatization needs to be evaluated. According to the evaluation results, the strengths and weaknesses of corporate accounting informatization can be determined and then the accounting informatization can be improved precisely. At present, research on accounting informatization evaluation has mostly focused on the construction of index systems and other theoretical aspects, and there are few case studies. In the related research of the construction of the accounting informatization index system, some scholars made appropriate innovations on the basis of previous research, but the index lacked advancement and did not conform to the current development trend of accounting informatization [10]. Or, the developments just followed the published evaluation index system of enterprise informatization [11]; for example, designs often deviated from the actual situation of the evaluated enterprises, resulting in more difficult data acquisition. At the same time, the design of the accounting informatization evaluation index system was based on the side description of the contents of accounting informatization, and lacked the research on accounting informatization itself. Such an index system cannot directly evaluate accounting informatization and lacks completeness and comprehensiveness. In the selection of evaluation methods, some scholars use the expert evaluation method to rate the weight of the index, which has great subjectivity [12]. In the process of quantifying the index, the quantitative grading method is adopted, in which the range of scores is also artificially prescribed, which makes the evaluation results lack objectivity [13].

In view of the above problems, this paper proposes an approach to evaluating accounting informatization based on intuitionistic fuzzy cross entropy. Firstly, the comprehensive evaluation index system of accounting informatization is constructed. In the index system, not only the portrayal of the accounting informatization, but also the influence of the new information technology such as artificial intelligence and XBRL (eXtensible Business Reporting Language) standardization on the accounting information is reflected. Secondly, the ratings given according to the evaluation index are in the form of linguistic terms, which is modeled by the intuitionistic fuzzy number. Because of their different professional knowledge and work experience, the evaluators’ understanding of the evaluation index and the company may be different. In the method of aggregating linguistic ratings, the weight of evaluators is taken into consideration. The consistency of ratings, which is measured by cross entropy, is used to determine the weight of evaluators. Smaller cross entropy means higher consistency; higher consistency leads to a greater weight, which can reduce the error caused by personal preference. Thirdly, the case study is conducted in detail. With the case study, the feasibility and practical application of the proposed approach is verified. It also provides a valuable reference for the implementation of evaluation of accounting informatization for other companies.

The structure of the rest of this paper is as follows: the second chapter reviews intuitionistic fuzzy sets, entropy, and cross entropy. The third chapter constructs the evaluation index from five aspects. The fourth chapter introduces the relative theory of intuitionistic fuzzy entropy and intuitionistic fuzzy cross entropy. The fifth chapter is a specific case study. The sixth chapter summarizes the paper.

## 2. Related Works

### 2.1. Intuitionistic Fuzzy Sets

Zadeh (1965) proposed the fuzzy set theory, which can be used to solve the fuzziness in the decision-making process. Based on the subjective thinking evaluation of the decision-makers, the concept of the degree of membership was proposed to describe the evaluation information [14]. After that, several extensions were developed including interval-valued fuzzy sets, intuitionistic fuzzy sets, two type-2 fuzzy sets, and so on. Atanassov (1986) proposed the concept of intuitionistic fuzzy sets (IFS) on the basis of fuzzy set theory. The intuitionistic fuzzy set is described by the degree of membership (belongingness), the degree of non-membership (non-belongingness), and degree of hesitation [15]. Since the uncertainty degree can be derived by the belongingness and non-belongingness, in most definitions only the non-belongingness and belongingness are used and the uncertainty degree is omitted.

**Definition** **1.** *The intuitionistic fuzzy set defined on a universe X is given as [16]:*

(1) $$A=\left\{\langle x,\mu_{A}\left(x\right),\nu_{A}\left(x\right)\rangle \left|x\in X\right|\right\}$$

The intuitionistic fuzzy number is the ordered pair of the degree of membership and the degree of non-membership of x in X to A. $\mu_{A}:X\to \left[0,1\right]$ and $\nu_{A}:X\to \left[0,1\right]$ represent the membership function and the non-membership function of the element x in X to A respectively, with the condition $0\leq \mu_{A}(x)+\nu_{A}(x)\leq 1$, ∀x∈X. In addition, $\pi_{A}(x)=1-\mu_{A}(x)-\nu_{A}(x)$ means the degree of hesitation of x to A, $0\leq \pi_{A}(x)\leq 1$. X represents intuitionistic fuzzy set A, then $\alpha =\left(\mu_{A}\left(x\right),\nu_{A}\left(x\right)\right)$ is the set of intuitionistic fuzzy numbers, named IFS(x).

Let $\alpha =\left(\mu_{A},\nu_{A}\right)$ and $\beta =\left(\mu_{B},\nu_{B}\right)$ be intuitionistic fuzzy numbers. Then, the operation rules of intuitionistic fuzzy numbers are as follows [16,17]:

$$\alpha +\beta =\left(\mu_{A}+\mu_{B}-\mu_{A}\mu_{B},\nu_{A}\nu_{B}\right);\;\alpha -\beta =\left(\mu_{A}\nu_{B},\nu_{A}+\mu_{B}-\nu_{A}\mu_{B},\right);\;\alpha \times \beta =\left(\mu_{A}\mu_{B},\nu_{A}+\nu_{B}-\nu_{A}\nu_{B}\right);\;\alpha ÷\beta =\left(\mu_{A}+\nu_{B}-\mu_{A}\nu_{B},\nu_{A}\mu_{B}\right);\;\lambda \alpha =\left(1-{\left(1-\mu_{A}\right)}^{\lambda },\nu_{A}{}^{\lambda }\right),\;\lambda >0;\;\alpha^{\lambda }=\left(\mu_{A}{}^{\lambda },1-{\left(1-\nu_{A}\right)}^{\lambda }\right),\;\lambda >0.$$

Some scholars proposed that the score function formula of the intuitionistic fuzzy number is $s\left(\alpha \right)=\mu_{\alpha }-\nu_{\alpha }$, s(α)∈[−1,1] [18,19]. Because the score function does not take the influence of the hesitation function $\pi_{\alpha }$ into account, the following score function formula is given.

**Definition** **2.** *Let $\alpha =\left(\mu_{\alpha },\nu_{\alpha }\right)$ is the intuitionistic fuzzy number. Then, its score function is given as [20]:*

(2) $$S\left(\alpha \right)=\mu_{\alpha }-\nu_{\alpha }+\frac{1-\mu_{\alpha }-\nu_{\alpha }}{2}$$

**Definition** **3.** *Let* $\alpha_{1}=\left(\mu_{\alpha_{1}},\nu_{\alpha_{1}}\right)$ *and* $\alpha_{2}=\left(\mu_{\alpha_{2}},\nu_{\alpha_{2}}\right)$ *be intuitionistic fuzzy numbers, and let* $S\left(\alpha_{1}\right)$ *and* $S\left(\alpha_{2}\right)$ *be scoring functions of* $\alpha_{1}$ *and* $\alpha_{2}$*, respectively. Then, we have [21]:*

If $S\left(\alpha_{1}\right)<S\left(\alpha_{2}\right)$, then $\alpha_{1}<\alpha_{2}$;

If $S\left(\alpha_{1}\right)=S\left(\alpha_{2}\right)$, then (1) if $\mu_{\alpha_{1}}+\nu_{\alpha_{1}}=\mu_{\alpha_{2}}+\nu_{\alpha_{2}}$, then $\alpha_{1}=\alpha_{2}$; (2) if $\mu_{\alpha_{1}}+\nu_{\alpha_{1}}<\mu_{\alpha_{2}}+\nu_{\alpha_{2}}$, then $\alpha_{1}<\alpha_{2}$.

**Definition** **4.** *Let* $\alpha_{j}=\left(\mu_{\alpha_{j}},\nu_{\alpha_{j}}\right)$*,* (j=1,2,…,n) *be a set of intuitionistic fuzzy numbers. Then, its weighted average operator function* f *is given as [16]:*

(3) $$f_{w}\left(\alpha_{1},\alpha_{2},\ldots ,\alpha_{n}\right)=\omega_{1}\alpha_{1}⊕\omega_{2}\alpha_{2}⊕\ldots ⊕\omega_{n}\alpha_{n}=\left(1-\prod_{j=1}^{n}{\left(1-\mu_{\alpha_{j}}\right)}^{\omega_{j}},\prod_{j=1}^{n}{\left(\nu_{\alpha_{j}}\right)}^{\omega_{j}}\right)$$

where $\omega ={\left(\omega_{1},\omega_{2},\ldots ,\omega_{n}\right)}^{T}$ is the weight vector of $\alpha_{j}$, with the condition that $0\leq \omega_{j}\leq 1$, j=1,2,…,n, and $\sum_{j=1}^{n}\omega_{j}=1$. In particular, if $\omega ={\left(\frac{1}{n},\frac{1}{n},\ldots ,\frac{1}{n}\right)}^{T}$, then f is the arithmetic mean operator of the intuitionistic fuzzy numbers.

### 2.2. Entropy and Cross Entropy

The uncertainty of intuitionistic fuzzy sets contains two aspects of intuition and fuzziness. The fuzzy information can be expressed by the absolute deviation of the degree of membership and the degree of non-membership, and the intuition information can be expressed by the degree of hesitation.

**Definition** **5.** *For any intuitionistic fuzzy set* A∈IFS(x)*, its intuitionistic fuzzy entropy formula is given as [22]:*

(4) $$E\left(A\right)=\frac{1}{n}\sum_{i=1}^{n}\frac{\min\left\{\mu_{A}\left(x_{i}\right),\nu_{A}\left(x_{i}\right)\right\}+\pi_{A}\left(x_{i}\right)}{\max\left\{\mu_{A}\left(x_{i}\right),\nu_{A}\left(x_{i}\right)\right\}+\pi_{A}\left(x_{i}\right)}$$

where n is the number of intuitionistic fuzzy numbers in the intuitionistic fuzzy set.

Kullback and Leibler (1951) put forward the concept of cross entropy as a way to measure the difference of information between two random vectors. The smaller the cross entropy, the higher the information consistency between the two random vectors [23].

**Definition** **6.** *For any intuitionistic fuzzy set* A,B∈IFS(x)*, its intuitionistic fuzzy cross entropy formula is given as [24]:*

(5) $$\begin{array}{c} D\left(A,B\right)=\sum_{i=1}^{n}\left[\mu_{A}\left(x_{i}\right)\ln\frac{\mu_{A}\left(x_{i}\right)}{\frac{1}{2}\left(\mu_{A}\left(x_{i}\right)+\mu_{B}\left(x_{i}\right)\right)}+\nu_{A}\left(x_{i}\right)\ln\frac{\nu_{A}\left(x_{i}\right)}{\frac{1}{2}\left(\nu_{A}\left(x_{i}\right)+\nu_{B}\left(x_{i}\right)\right)}\right]+\\ \sum_{i=1}^{n}\left[\mu_{B}\left(x_{i}\right)\ln\frac{\mu_{\beta }\left(x_{i}\right)}{\frac{1}{2}\left(\mu_{A}\left(x_{i}\right)+\mu_{B}\left(x_{i}\right)\right)}+\nu_{B}\left(x_{i}\right)\ln\frac{\nu_{B}\left(x_{i}\right)}{\frac{1}{2}\left(\nu_{A}\left(x_{i}\right)+\nu_{B}\left(x_{i}\right)\right)}\right] \end{array}$$

## 3. Construction of the Evaluation Index System of Enterprise Accounting Information

Considering the content of enterprise accounting informatization evaluation, the achievements of existing enterprise informatization and accounting informatization as well as the basic index structure of national enterprise informatization, using methods of theoretical analysis and experience selection, the evaluation index system of enterprise accounting informatization is constructed from five aspects: strategic position B1, infrastructure construction B2, accounting informatization implementation B3, informatization guarantee B4, and application benefits B5, to evaluate the impact of accounting informatization construction on the enterprise accounting informatization level in the process of enterprise accounting informatization, as is shown in Table 1.

### 3.1. Evaluation Attributes of Strategic Position

(1) Recognition and participation

① High-level leaders’ importance

In order to carry out the accounting informatization, we first need the attention and support of high-level leaders. Ideologically, it is recognized that accounting information is an important part of enterprise information construction, and it helps to improve the accounting management ability and decision-making level. Accounting information will be guaranteed in the organization and management, departmental organization, and personnel [25].

② Proficiency of the leadership in the application of the accounting information system

This index reflects the application level of top leaders to accounting informatization. The leadership should not only know what the accounting informatization is “doing”, but also know how to do it in detail. Therefore, in addition to having the ability to deal with accounting business, the computer-related skills also need to be mastered to apply an accounting information system [26].

(2) Accounting informatization budget and planning

① The construction of accounting informatization and the suitability of the operating environment

The index reflects the integrating degree of the accounting information system and the operating environment. The higher fit is beneficial to the optimal allocation of resources, and it can make the management of the enterprise reach the effect of “1 + 1 > 2” [26].

② Accounting informatization budget or planning and actual completion

The information budget/planning in “The Basic Index Composition Scheme and Measurement Method of Chinese Enterprise Informatization” can be divided into high, intermediate, and low levels. In the high level, the accounting information budget/planning is written separately, and the detailed budget/planning for the software development level and development cost is made. In the intermediate level, accounting informatization is scattered in the overall budget/planning, and is included in the construction of enterprise informatization. In the low level, there is no written accounting information budget/planning [27].

(3) Strategy and investment structure

① Investment in accounting informatization

This index reflects the financial support for the construction of accounting informatization. After meeting the normal production activities, the funds of small and medium-sized enterprises will be invested in those projects with fast return and high rate of return on investment [28]. Also, the construction of accounting informatization is a long-term process. Under the limited conditions of capital, the investment of accounting informatization will change according to the actual operation of the enterprise. The higher the degree of satisfaction of accounting informatization construction to investment funds, the smaller the funding gap, and the greater the support.

② Investment structure of accounting informatization

This index is used to evaluate the rationality of the accounting informatization investment structure [29]. The investment of enterprises for accounting informatization construction is limited, but the direction of investment involves the construction of hardware and software facilities, later maintenance and software upgrading, daily management, information resources, and many other aspects [30]. Only a reasonable proportion of investment can contribute to the coordinated development of accounting information system construction, maintenance, updating, and information resources.

### 3.2. Evaluation Attributes of Infrastructure Construction

(1) Technical route of system architecture

① The unification of the accounting information system and the overall system architecture technology roadmap

The purpose of accounting informatization construction is to rely on the integration of the accounting information system, so that the accounting information can be shared and concentrated. When enterprises are developing to the maturity stage, business scope and scale will increase, and accounting information will have a wider scope and require a higher concentration [31]. The construction of accounting information should be carried out under the unified leadership of top-level design, and maintain the consistency of the accounting information system and the overall system architecture [25].

② Extensibility of the accounting information system architecture

The enterprise accounting informatization is constructed on the basis of unified technical standards, coding rules, and system parameters, in which the function can be built by module one by one, and the range can be covered from local to comprehensive. The extensibility of the accounting information system architecture gives flexibility to the construction of accounting informatization [31].

(2) Level of infrastructure construction

① Data integration of the accounting information system

The index is used to evaluate the integration degree between the internal financial system and other systems. One of the trends of informatization is the integration of different business systems. Data integration in different application systems can eventually realize the unified operation of data and reduce the cost of the unified format. The focus will be on the integration of the financial system with the business system, eliminating redundant operating procedures, effectively reorganizing the accounting business, and improving the processing speed and accuracy of the enterprise accounting business [25].

② Construction of a financial sharing service platform

This index is used to evaluate the construction of a financial shared service platform. The financial sharing service platform can carry out the intensive processing of the financial services of different departments, especially for economic businesses with a large business volume and repeated processing modes. The financial sharing service platform covers the modules of data collection, inspection, exchange, storage, inquiry, and analysis [32]. It has a general transaction processing module, a decision support function, and an expert consultation function [33], providing standard sharing services for different regions’ organizational structures.

### 3.3. Evaluation Attributes of Accounting Informatization Implementation

(1) Informatization level of the process of accounting information movement

① Informatization of the accounting information generation process

The generation of accounting data directly affects the reliability and usefulness of the accounting information. The integration of the business system and financial system affects the efficiency of the accounting data collection [32]. The level of informatization of the accounting information generation process can be evaluated by the integration degree of the financial business system, the degree of coordination of the financial business processing, the degree of the automatic transfer, and the collection rate of the data automation.

② Informatization of the accounting information audit process

The main influence of accounting informatization on auditing is to enhance the logicality and systematization of the accounting system and weaken the control relationships among accounts. It is necessary to establish the information management system of the accounting audit process, improve the mechanism of audit risk prevention [34], and realize the intelligence of the audit through the combination of Internet Audit, cloud audit, and large data audit, as well as improve the informatization degree of the audit link [35].

③ Informatization of the accounting information exchange process

The important premise of information exchange is the standardization of accounting information, which emphasizes the exchange of data format, and requires a standard system construction, including metadata standards, XBRL general classification standards [36], and so on, in order to standardize accounting information so as to meet the needs of different information users. At the same time, accounting information exchange also needs data system integration support to realize data sharing among different departments [37].

④ Informatization of the accounting information mining process

The link of accounting information mining can be considered as the process of reprocessing of accounting information. The information concealing in the accounting data which is not used is analyzed in a deeper and systematic level using methods of classification, cluster, association grouping, and complex data mining, and the data information is converted into knowledge resources to increase the value of the accounting information [38].

⑤ Informatization of the accounting information use process

The use of accounting information refers to the direct acquisition and use of accounting information before and after the addition of value. The application of accounting information can improve the frequency of information use and the exchange rate of accounting information as well as increase the value of the use of accounting information [38].

(2) Accounting decision informatization

The index of the informatization degree of accounting strategy decisions is used to evaluate the level to which the application of accounting informatization supports accounting strategic decision-making [39]. Accounting strategic decision-making refers to the accounting decisions made by high-level decision-makers that have a significant impact on the future development of enterprises. Enterprises in the phase of financial management informatization should gradually realize financial analysis, comprehensive budget management, risk control, performance assessment, and other decision support informatization. Accounting tactical decision-making refers to specific accounting decisions that are implemented within the scope of an organization, and references specific decisions of the strategic decision-making process. Accounting business decision-making denotes decisions made with the aim of improving production efficiency and daily work efficiency in executing the plan, which only affects the local area.

### 3.4. Evaluation Attributes of Informatization Guarantee

(1) Safety and reliability

① Construction of operation and maintenance management system

The index is used to evaluate the construction of the IT operation and maintenance management system. The main points of the evaluation include the construction of the system operation and maintenance management mechanism, the change and release management of the information system, the continuity management of the information system, the management of health inspections, and the construction of the operation and maintenance capacity [40].

② Informatization security management system

This index appraises the construction of the enterprise information security management system. The infrastructure of informatization is the most basic requirement of the construction of accounting information, and involves the creation of a safety management system to ensure its security, including an information security organization [41], an information security management system [42], and an information system security operation control level [13]. The purpose of this infrastructure is to operate and make improvements in accordance with the four important links of planning, implementation, inspection, and improvement, as well as to ensure the comprehensive, quality management of the enterprise information security management system construction process to ensure the effectiveness of the implementation of accounting informatization [39].

③ Safety management of accounting informatization personnel

Informatization security needs not only standard specification, but also support from accounting informatization talents. The index is used to evaluate the security and reliability of information from the viewpoint of personnel. Enterprises should pay attention to the employment and examination of important and sensitive posts, the safety awareness education of accounting informatization personnel, the management of external personnel, and the personnel confidentiality management [41].

④ Application degree of new technology of accounting informatization

The index mainly reflects the application of new technologies such as block chain, big data, Artificial Intelligence, and cloud computing in accounting informatization [43]. The integration of large data and traditional business can carry out business innovation and establish financial analysis tools quickly; the combination of artificial intelligence and finance can produce intelligent finance; cloud accounting combines the advantages of Internet information technology and provides convenience for the collection and storage of accounting information by using large data and virtualization [44].

(2) Human resources

① Penetration rate of accounting informatization technology

This index mainly evaluates the penetration of the application of informatization technology in accounting informatization personnel [45]. Accounting audit and related personnel should not only master the professional knowledge in the field of finance and accounting, but also pay more attention to the training of information technology skills so as to carry out the informatization work of accounting audits as smoothly as possible.

② Coverage of accounting informatization training

The index evaluation refers to the training of enterprise accounting informatization. The function of the training is to shorten the distance between the present working level and the frontier knowledge of the information personnel, and provide the necessary conditions and guidance for the growth of talents [46]. Expanding training coverage and paying attention to improve the efficiency of accounting informatization training can play a role in guiding employees to improve their professional quality and skill level.

③ Policy of accounting informatization personnel

The construction of enterprise accounting informatization needs compound talents who are not only familiar with the accounting auditing standards system and the internal control standard system, but also understand accounting informatization [47]. In the personnel policy, we should establish a mechanism for training, using, introducing, selecting, and evaluating talents, focusing on attracting scientific and technical personnel in colleges and universities, professionals with the background of overseas work, and informational talents with rich experience [48].

### 3.5. Evaluation Attributes of Application Benefits

(1) Economic benefits

① Direct economic benefits

The index is used to evaluate the direct economic benefits brought by the application of accounting informatization for enterprises, which can be reflected in the promotion of profitability, solvency, operation, labor productivity, and asset quality [49]. Moreover, the construction of accounting informatization improves the accuracy of the collection, sorting, and analysis of the accounting information, and improves the speed of the financial accounting [50].

② Indirect economic benefits

This index evaluates the indirect economic benefits brought by the application of accounting informatization from three perspectives: enterprise customers, internal processes, and learning and growth. Accounting informatization helps to build good customer relationships and creates an atmosphere that supports informatization and the sustainable development of enterprises [51]. Accounting informatization causes the focus of work to shift from daily accounting affairs to management decision-making, such as business process optimization and organizational structure adjustment. This shift of the center of gravity of work can lead enterprises to obtain indirect economic benefits [52].

(2) Management benefits

① Financial management informatization

This index mainly evaluates the impact of the application of accounting informatization on financial management. Accounting informatization makes enterprises develop from accounting informatization to financial management informatization gradually, and can provide decision-making support for financial analysis, risk control, comprehensive budget management, performance assessment, and other management accounting modules [53].

② Internal control process informatization

An important part of the internal control system is accounting control. Accounting information is the means of internal control for supervision and communication. At the same time, the feedback mechanism of internal control is the prerequisite for accounting information to play a role. In the information environment, the key controls, such as organization management control, system development control, key operation control, and data and program control, are integrated into the accounting information system to ensure the quality of the accounting information [26].

(3) Social benefit

① Save resources

Promoting enterprise accounting informatization construction can save social resources. One of the values and goals of informatization is to replace manual work and paper documents [25]. The paperless development of accounting information can make business transactions free from the limitations of paper documents. At the same time, the application of accounting informatization can save energy and reduce consumption, eliminate backward productivity, and optimize the allocation of resources, thus reducing the cost of information supply and losses, protecting the environment, and saving social resources.

② Social progress

Promoting the construction of enterprise accounting informatization depends on the progress of modern science and technology. The application of accounting informatization can improve the management level of enterprises and enhance the core competitiveness of enterprises [13]. At the same time, it is beneficial to gradually cultivate an accounting information service industry with high-quality service, a good social reputation, and broad prospects for development by providing hardware and software products, technical services, and consulting services for enterprises and institutions.

## 4. Method to Deal with the Accounting Informatization Linguistic Evaluation

Suppose that the intuitionistic fuzzy numbers $r_{tj}^{nk}=\left(p_{tj}^{nk},q_{tj}^{nk}\right)$ and $r_{lj}^{nk}=\left(p_{lj}^{nk},q_{lj}^{nk}\right)$ are the values used to calculate the weight of the index t and the weight of the evaluation value l, given by the evaluators $D_{k}\in D$, with respect to the index j at the nth level. Let $A^{nk}=\left\{r_{t1}^{nk},r_{t2}^{nk},\ldots ,r_{tm}^{nk}\right\}$ be the intuitionistic fuzzy set of the nth level.

The procedures are given as follows.

Step 1: According to the Table 2, transforming the weight of the index and evaluation value language assessment sets to the basic intuitionistic fuzzy sets $A^{nk}=\left\{r_{t1}^{nk},r_{t2}^{nk},\ldots ,r_{tm}^{nk}\right\}$ and $B^{nk}=\left\{r_{l1}^{nk},r_{l2}^{nk},\ldots ,r_{lm}^{nk}\right\}$.

(1) Determine the weight of the evaluators based on the weights of the index.

Step 2: Determine the individual evaluation results $\gamma_{t}^{nk}$ on the weight of the index of evaluator k, with respect to the index at the nth level. Based on the intuitionistic fuzzy set of the weights of the index $A^{nk}=\left\{r_{t1}^{nk},r_{t2}^{nk},\ldots ,r_{tm}^{nk}\right\}$ and the initial index weight vector $\omega_{t}={\left(\omega_{t1},\omega_{t2},\ldots ,\omega_{tm}\right)}^{T}$, which is the average of the ratings given by evaluators, the weighted average operator of the intuitionistic fuzzy numbers can be used to obtain the intuitionistic fuzzy set $Y_{t}^{n}=\left\{\gamma_{t}^{n1},\gamma_{t}^{n2},\ldots ,\gamma_{t}^{ns}\right\}$.

(6) $$\gamma_{t}^{nk}=\overset{m}{\underset{j=1}{⊕}}r_{tj}^{nk}\omega_{tj}=\left(\mu_{t}^{nk},\nu_{t}^{nk}\right)\;\mu_{t}^{nk}=1-\prod_{j=1}^{m}{\left(1-p_{tj}^{nk}\right)}^{\omega_{tj}},\;\nu_{t}^{nk}=\prod_{j=1}^{m}{\left(q_{{}_{tj}}^{nk}\right)}^{\omega_{tj}},\;k=1,2,\ldots ,s,\;j=1,2,\ldots ,m$$

The initial index weight vector is the average of the ratings given by the evaluators without considering the weight of the evaluators.

Step 3: Compute the entropy weight $e_{t}^{nk}$ on the weight of the index of evaluator k, with respect to the index at the nth level, based on the entropy $E_{t}^{nk}$ of the intuitionistic fuzzy set.

(7) $$E_{t}^{nk}=\frac{\min\left\{\mu_{t}^{nk},\nu_{t}^{nk}\right\}+\pi_{t}^{nk}}{\max\left\{\mu_{t}^{nk},\nu_{t}^{nk}\right\}+\pi_{t}^{nk}},\;k=1,2,\ldots ,s,\;j=1,2,\ldots ,m$$

(8) $$e_{t}^{nk}=\frac{1-E_{t}^{nk}}{s-\sum_{k=1}^{s}E_{t}^{nk}},\;k=1,2,\ldots ,s$$

Step 4: Compute the cross entropy $D\left(\gamma_{t}^{nf},\gamma_{t}^{ng}\right)$ of the evaluator f relative to the evaluator g, with respect to the index in the nth level. Compute the $E\left(\gamma_{t}^{nk}\right)$ of evaluator k relative to all the other evaluators.

(9) $$\begin{array}{c} D\left(\gamma_{t}^{nf},\gamma_{t}^{ng}\right)=\mu_{{}_{t}}^{nf}\ln\frac{\mu_{t}^{nf}}{\frac{1}{2}\left(\mu_{t}^{nf}+\mu_{t}^{ng}\right)}+\nu_{t}^{nf}\ln\frac{\nu_{t}^{nf}}{\frac{1}{2}\left(\nu_{t}^{nf}+\nu_{t}^{ng}\right)}+\\ \mu_{t}^{ng}\ln\frac{\mu_{t}^{ng}}{\frac{1}{2}\left(\mu_{t}^{nf}+\mu_{t}^{ng}\right)}+\nu_{t}^{ng}\ln\frac{\nu_{t}^{ng}}{\frac{1}{2}\left(\nu_{t}^{nf}+\nu_{t}^{ng}\right)} \end{array},\;f\neq g,\;f,g\in \left[1,s\right]$$

(10) $$E\left(\gamma_{t}^{nk}\right)=\sum_{g=1}^{s}D\left(\gamma_{t}^{nk},\gamma_{t}^{ng}\right),\;k\neq g,\;g=1,2,\ldots ,s$$

Step 5: Compute the cross entropy weight $r_{t}^{nk}$ of evaluator k in relation to the weight of the index, with respect to the index at the nth level.

(11) $$r_{t}^{nk}=\frac{\frac{1}{E\left(\gamma_{t}^{nk}\right)}}{\sum_{k=1}^{s}\frac{1}{E\left(\gamma_{t}^{nk}\right)}},\;0\leq r_{k}\leq 1,\;k=1,2,\ldots ,s$$

Step 6: Calculate the comprehensive weight $\eta_{t}^{nk}$ in relation to the weight of the index at the nth level, which synthesizes the initial evaluator weight $\lambda_{k}$, entropy weight $e_{t}^{nk}$, and cross entropy weight $r_{t}^{nk}$.

(12) $$\eta_{t}^{nk}=\alpha \lambda_{k}+\beta e_{t}^{\mathrm{nk}}+\theta r_{t}^{\mathrm{nk}},\;k=1,2,\ldots ,s,\;\alpha +\beta +\theta =1$$

(2) Determine the weight of the evaluators based on the evaluation value.

Step 7: Determine the individual evaluation results $\gamma_{t}^{nk}$ on the evaluation value of evaluator k, with respect to the index at the nth level. Based on the intuitionistic fuzzy set of the evaluation value $B^{nk}=\left\{r_{l1}^{nk},r_{l2}^{nk},\ldots ,r_{lm}^{nk}\right\}$ and the initial index weight vector $\omega_{l}={\left(\omega_{l1},\omega_{l2},\ldots ,\omega_{lm}\right)}^{T}$, use the weighted average operator of the intuitionistic fuzzy numbers to obtain the intuitionistic fuzzy set $Y_{l}^{nk}=\left\{\gamma_{l}^{n1},\gamma_{l}^{n2},\ldots ,\gamma_{l}^{ns}\right\}$.

(13) $$\gamma_{l}^{nk}=\overset{m}{\underset{j=1}{⊕}}r_{j}^{nk}\omega_{lj}=\left(u_{l}^{nk},\nu_{l}^{nk}\right)\;\mu_{l}^{nk}=1-\prod_{j=1}^{m}{\left(1-p_{lj}^{nk}\right)}^{\omega_{lj}},\;\nu_{l}^{nk}=\prod_{j=1}^{m}{\left(\nu_{{}_{lj}}^{nk}\right)}^{\omega_{lj}},\;k=1,2,\ldots ,s,\;j=1,2,\ldots ,m$$

Step 8: Compute the entropy weight $e_{l}^{nk}$ on the evaluation value of evaluator k, with respect to the index at the nth level, based on the entropy $E_{l}^{nk}$ of the intuitionistic fuzzy set.

(14) $$E_{l}^{nk}=\frac{\min\left\{\mu_{l}^{nk},\nu_{l}^{nk}\right\}+\pi_{l}^{nk}}{\max\left\{\mu_{l}^{nk},\nu_{l}^{nk}\right\}+\pi_{l}^{nk}},\;k=1,2,\ldots ,s,\;j=1,2,\ldots ,m$$

(15) $$e_{l}^{nk}=\frac{1-E_{l}^{nk}}{s-\sum_{k=1}^{s}E_{l}^{nk}},\;k=1,2,\ldots ,s$$

Step 9: Compute the cross entropy $D\left(\gamma_{l}^{nf},\gamma_{l}^{ng}\right)$ of evaluator f relative to evaluator g, with respect to the index at the nth level. Compute the $E\left(\gamma_{l}^{nk}\right)$ of evaluator k relative to all the other evaluators.

(16) $$\begin{array}{c} D\left(\gamma_{l}^{nf},\gamma_{l}^{ng}\right)=\mu_{l}^{nf}\ln\frac{\mu_{l}^{nf}}{\frac{1}{2}\left(\mu_{l}^{nf}+\mu_{l}^{ng}\right)}+\nu_{l}^{nf}\ln\frac{\nu_{l}^{nf}}{\frac{1}{2}\left(\nu_{l}^{nf}+\nu_{l}^{ng}\right)}+\\ \mu_{l}^{ng}\ln\frac{\mu_{l}^{ng}}{\frac{1}{2}\left(\mu_{l}^{nf}+\mu_{l}^{ng}\right)}+\nu_{l}^{ng}\ln\frac{\nu_{l}^{ng}}{\frac{1}{2}\left(\nu_{l}^{nf}+\nu_{l}^{ng}\right)} \end{array},\;f\neq g,\;f,g\in \left[1,s\right]$$

(17) $$E\left(\gamma_{l}^{nk}\right)=\sum_{g=1}^{s}D\left(\gamma_{l}^{nk},\gamma_{l}^{ng}\right),\;k\neq g,\;g=1,2,\ldots ,s$$

Step 10: Compute the cross entropy weight $r_{l}^{nk}$ of evaluator k in relation to the evaluation value, with respect to the index at the nth level.

(18) $$r_{l}^{nk}=\frac{\frac{1}{E\left(\gamma_{l}^{nk}\right)}}{\sum_{k=1}^{s}\frac{1}{E\left(\gamma_{l}^{nk}\right)}},\;0\leq r_{k}\leq 1,\;k=1,2,\ldots ,s$$

Step 11: Calculate the comprehensive weight $\eta_{l}^{k}$ in relation to the evaluation value, which synthesizes the initial evaluator weight $\lambda_{k}$, entropy weight $e_{l}^{nk}$, and cross entropy weight $r_{l}^{nk}$.

(19) $$\eta_{l}^{nk}=\alpha \lambda_{k}+\beta e_{l}^{nk}+\theta r_{l}^{nk},\;k=1,2,\ldots ,s,\;\alpha +\beta +\theta =1$$

(3) Determine the evaluation value.

Step 12: Compute the intuitionistic fuzzy set of the weighted weight of the index $\alpha_{j}^{n}$, with respect to the index j at the nth level. Use the weighted average operator formula to combine the comprehensive weight $\eta_{t}^{nk}$ and the intuitionistic fuzzy weight of the index $r_{tj}^{nk}=\left(p_{tj}^{nk},q_{tj}^{nk}\right)$ at the nth level.

(20) $$\alpha_{j}^{n}=\overset{s}{\underset{k=1}{⊕}}r_{tj}^{nk}\eta_{t}^{nk}=\left(\mu_{tj}^{n},\nu_{tj}^{n}\right)\;\mu_{tj}^{n}=1-\prod_{k=1}^{s}{\left(1-p_{tj}^{nk}\right)}^{\eta_{t}^{nk}},\;\nu_{tj}^{n}=\prod_{k=1}^{s}{\left(q_{{}_{tj}}^{nk}\right)}^{\eta_{t}^{nk}},\;j=1,2,\ldots ,m,\;k=1,2,\ldots ,s$$

Step 13: Compute the intuitionistic fuzzy set of the weighted evaluation value $\beta_{j}^{n}$, with respect to the index j at the nth level. Use the weighted average operator formula to combine the comprehensive weight $\eta_{l}^{k}$ and the intuitionistic fuzzy number of the evaluation value $r_{lj}^{nk}=\left(p_{lj}^{nk},q_{lj}^{nk}\right)$ at the nth level.

(21) $$\beta_{j}^{n}=\overset{s}{\underset{k=1}{⊕}}r_{lj}^{nk}\eta_{l}^{k}=\left(\mu_{lj}^{n},\nu_{lj}^{n}\right)\;\mu_{lj}^{n}=1-\prod_{k=1}^{s}{\left(1-p_{lj}^{nk}\right)}^{\eta_{l}^{nk}},\;\nu_{lj}^{n}=\prod_{k=1}^{s}{\left(q_{{}_{lj}}^{nk}\right)}^{\eta_{l}^{nk}},\;j=1,2,\ldots ,m,\;k=1,2,\ldots ,s$$

Step 14: Combine the intuitionistic fuzzy number of the weighted weight of $\alpha_{j}^{n}$ and the intuitionistic fuzzy number of the weighted evaluation value $\beta_{j}^{n}$ to obtain the intuitionistic fuzzy set of the comprehensive evaluation value $\beta_{}^{n-1}$ at the n−1th level by weighted average.

(22) $$\beta_{}^{n-1}=\frac{\sum_{j=1}^{m}\alpha_{j}^{n}⊗\beta_{j}^{n}}{\sum_{j=1}^{m}\alpha_{j}^{n}},\;j=1,2,\ldots ,m$$

Step 15: Regard the comprehensive evaluation value intuitionistic fuzzy number $\beta_{}^{n-1}$ as a weighted evaluation value intuitionistic fuzzy number at the n−1th level. When the evaluation results of the parent level index are required, repeat step 13 until the evaluation results in an intuitionistic fuzzy number of the first level index.

Step 16: Convert the intuitionistic fuzzy weight of index $\alpha_{j}^{n}=\left(\mu_{tj}^{n},\nu_{tj}^{n}\right)$ and the intuitionistic fuzzy number of the evaluation value $\beta_{j}^{n}=\left(\mu_{lj}^{n},\nu_{lj}^{n}\right)$ to score the function value.

(23) $$S\left(\alpha_{tj}^{n}\right)=\mu_{tj}^{n}-\nu_{tj}^{n}+\frac{1-\mu_{tj}^{n}-\nu_{tj}^{n}}{2},\;j=1,2,\ldots ,m$$

(24) $$S\left(\beta_{lj}^{n}\right)=\mu_{lj}^{n}-\nu_{lj}^{n}+\frac{1-\mu_{lj}^{n}-\nu_{lj}^{n}}{2},\;j=1,2,\ldots ,m$$

## 5. Application of Accounting Informatization Evaluation

This section builds on the established accounting informatization evaluation system and evaluation method, which uses a questionnaire survey to collect the perceptions of staff on the current status of accounting informatization and accounting information technology of company A to evaluate the company’s accounting informatization level. There are 30 valid questionnaires that can be applied to the calculation and analysis in this questionnaire survey. From the valid questionnaire, we can see that there is a subjective bias in individual evaluation values. Scores in some questionnaires are very high or very low. The use of the consistency of individuals and groups measured by cross entropy can deduce the influence caused by personal bias.

Company A was approved and established in 2014. It is mainly engaged in computer software and hardware, network technology development, technology promotion, technical services, and the sale of self-developed software products. Company A belongs to the emerging service industry relying on the Internet. Company A began to use U8 management software in 2014 when the company was incorporated, and the office automation module was developed in April 2017 and put into use in June.

### 5.1. Calculate the Results

(1) Calculate the evaluator weights

In the implementation, the initial weight of the evaluator, the weight of the entropy, and the weight of the cross entropy need to be determined. Since the initial weight is given manually, this value is more subjective. In order to decrease the bias of subject weights, the initial weight is given a lower weight and the other two kinds of weights are given higher weights. The weight of the evaluator, the weight of the entropy, and the weight of the cross entropy are respectively weighted to 0.1, 0.4, and 0.5, by steps 1–6. Based on the evaluator’s score on the index’s weight, and the evaluator’s rating on the performance of each company’s index, the weights of index, and the weights of the evaluation values are shown in Table 3.

(2) Evaluation result calculation

Combining the evaluator weights, through the steps 1–15, calculate the weighted weight index, the weighted evaluation value, and the final performance of Company A. In order to facilitate the analysis and comparison of various indexes, Formula (16) is used to calculate the score function of each intuitionistic fuzzy number, and the score function value is used to represent the importance, which is shown in Table 4.

### 5.2. Analyze the Results

(1) Results of the comprehensive evaluation of the third-level index

**Figure 1:**
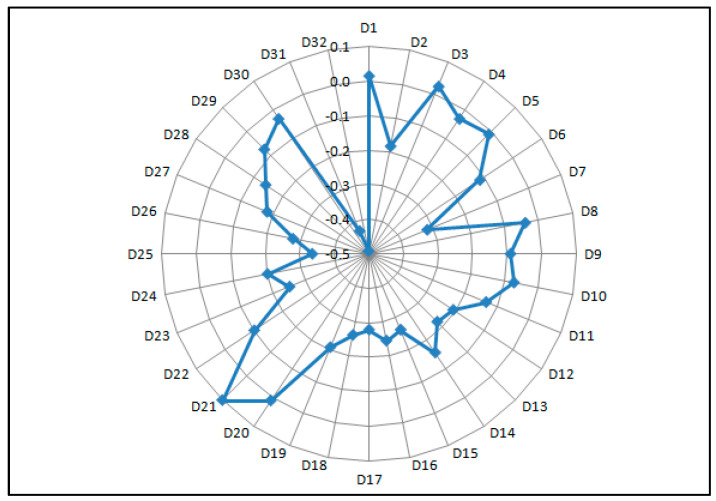
Evaluation value of the third-level attributes.

Figure 1 is a radar chart based on the comprehensive evaluation value of the third-level index.

It can be seen that the comprehensive evaluation values of D_31_ and D_32_ are the lowest, because the evaluators subjectively believe that the social benefit index is not significant. Company A is a technology-oriented enterprise, and currently relies on the application of information technology research and development and accounting information technology to improve the efficiency of resource uses and reduce operating costs. Therefore, the comprehensive evaluation value of D_31_ is slightly higher than that of D_32_. D_25_ and D_26_ are also the same, indicating that the importance of indexes has a great impact on the overall evaluation value. However, Company A seizes talent resources through high-paying, high-welfare talent policies, not only for technical talents in data development, algorithm writing, and server R&D, but also for informatization talents such as data mining and investment analysis. Therefore, the comprehensive evaluation value of D_26_ is slightly higher than that of D_25_.

On the contrary, indexes with higher comprehensive evaluation values include D_21_, D_3_, D_1_, D_20_, D_5_, and D_30_. Company A has done a better job in the information security management system than in personnel safety management. It is relatively perfect in information security organization, information security management system, and information system security operation control level. In addition, the integration of the accounting informatization with the current stage of Company A’s development, the scale of the company, internal culture, organizational structure, and the overall quality of employees is highly consistent. Senior leaders attach importance to the work of accounting informatization. They have set up accounting informatization positions and designated specialized positions to take charge of the daily development of accounting informatization work. In the current accounting information construction environment, although Company A did not separate accounting information into separate articles, it formulated a more detailed plan for the construction of accounting information and strived to use the advanced nature of the accounting information system to accelerate the accounting work. In addition, key controls such as the organizational management control, system development control, key operation control, and data and program control are integrated into the accounting information system, which improves the effectiveness of the design and operation of the internal control specification system.

(2) Analysis of the comprehensive evaluation value of the third-level index considering importance

Comparing the ranking of comprehensive evaluation values with the ranking of indexes, rankings with a large decline in comprehensive evaluation values include: D_13_, D_15_, and D_17_. The importance of these indexes is in a medium position, but because the evaluation value of these indexes is too low, the comprehensive evaluation value combined with importance is at a lower level. By analyzing the information process of accounting information, the audit process informatization and mining processes are low, so Company A has not obtained the evidence related to the control and assurance measures of the accounting information system through IT audit to realize the control of the accounting informatization risk. At the same time, the data mining degree of accounting information is not enough, and the value of the accounting information cannot be increased. The evaluation value of the accounting strategic decision-making information is relatively low, indicating that high-level decision-makers do not use the accounting information to make accounting decisions, which has a significant impact on the future development of the company. Or, this low value may be because the evaluators are limited by their own scope of work and unable to know the specific circumstances, and as such give a lower score.

The rankings for the overall evaluation value increase are: D_21_, D_30_, and D_9_. The weight of these indexes is in the middle-lower position, but due to the high evaluation value of these indexes, the comprehensive evaluation value with combined importance is at a higher level. This shows that the company’s accounting information system architecture is highly scalable, giving the group some flexibility in its informatization. The comprehensive evaluation of the remaining two indexes has been given in the previous paragraph.

(3) Analysis of the results of the comprehensive evaluation of the third-level indexes under the secondary indexes

From the perspective of the third-level indexes corresponding to the second-level indexes, indexes related to C_8_ are ranked in descending order of comprehensive evaluation values as follows: D_21_, D_20_, D_22_, and D_23_. At present, Company A attaches great importance to safety and reliability. With the help of information technology, it has made great efforts in the establishment of an information security management system, an operation and maintenance management system, and hardware environment, and has gradually begun to increase the training of accounting information personnel security awareness and information technology skills. The company strives to provide double protection for the safety and reliability of accounting informatization from both technical and personnel aspects.

The third-level indexes related to C_6_ of the informatization level in the process of accounting information movement are ranked in descending order of comprehensive evaluation values as follows: D_14_, D_12_, D_13_, D_16_, and D_15_. Company A is currently working on the integration of financial business systems, which is conducive to the improvement of the information exchange process of accounting information. The informatization of accounting information generation process can provide the most original accounting information through the accounting informatization stage, and the accounting information mining, use, and auditing process informatization require the support of higher-level accounting decision support systems and computer auditing systems, so the level of informatization is slightly lower.

The sub-indexes of C_2_ are ranked in descending order of comprehensive evaluation values as follows: D_3_, D_5_, and D_4_. Company A has developed rapidly; the management model is not yet defined, the business is relatively simple, and accounting information needs diversity. Therefore, in the promotion of accounting informatization in enterprises, the importance of information systems, enterprise management models, and business processes has been considered.

In the aspect of accounting decision informatization (C_7_), the sub-indexes are ranked in descending order as follows: D_19_, D_18_, and D_17_. Although the importance level of accounting decision-making informatization is the highest, the level of informatization of business decisions in Company A accounting practice is the highest. Interviewees are mostly grass-roots financial personnel. Compared with general strategic decisions, most information workers are exposed to information-based business decision-making.

The company performance is better in D_1_ than D_2_ of recognition and participation (C_1_). When senior management of company A promotes information construction within the company, it only provides support for the development of accounting informatization, and does not personally participate in the application of accounting information systems. The accounting information system’s development level, integration scale and other construction contents are still the responsibility of the accounting director.

With respect to the index C_11_, the rank of corresponding sub-index in descending order is D_30_ and D_29_. The information management of financial management is the goal pursued by company A, and the level of informatization of internal control processes is relatively higher. Because Company A builds and perfects the internal control feedback mechanism, it integrates key control points into the accounting information system to ensure the quality of accounting information.

In technical route of system architecture (C_4_), the company performance is better in D_8_ than D_9_. Company A maintains the consistency of the technical route between the accounting information system and the enterprise management system to achieve integration between various systems. Due to the limitation of the company’s own business characteristics, it is not necessary to develop technology companies and asset-light assets, and it is not necessary to develop enterprise groups with a wide geographical distribution and complex business processes. Therefore, the information technology system has a low level of information scalability.

The indexes related to the three levels of C_5_ are ranked in descending order of comprehensive evaluation values as follows: D_10_ and D_11_. Company A is currently working on the data integration of the accounting information system to achieve financial business integration so as to facilitate the improvement of accounting information exchange and increase the use frequency to achieve information sharing. The progress of the construction of the financial sharing service platform is relatively slow.

The comprehensive evaluation value of the third-level indexes related to C_3_ from high to low is in the order of D_6_, D_7_. In the construction of the accounting information of Company A, sufficient financial support has been provided, but the rationality of the investment structure is not yet considered comprehensively. For example, the form of entrusting an external unit to carry out accounting software development is relatively expensive for commissioned development.

The sub-indexes of C_10_ are ranked in descending order of comprehensive evaluation values as follows: D_28_ and D_27_. Company A is still in a loss situation; the commercialization process is slow, and it is still exploring a mature profit model. Therefore, the accounting information is not reflected in the direct economic interests, but reflected in improving the indirect economic benefits such as accounting speed and cost control.

The indexes which are at the sub-level of C_9_ are ranked in descending order of comprehensive evaluation values as follows: D_24_, D_26_, and D_25_. Company A has gradually begun to increase the intensity of staff training and—through the training of information technology aspects and skills—strengthen the training of accounting information technology personnel.

The third-level indexes related to C_12_ are ranked in descending order of comprehensive evaluation values as follows: D_31_ and D_32_. Company A is an asset-light enterprise that focuses on technology research and development. The main research and development expenses are reflected in IT staff remuneration, daily energy consumption, and other aspects. The reduction in R&D expenses will reduce energy consumption and optimize resource allocation.

### 5.3. Suggestions

First, we must vigorously promote the progress of infrastructure construction. Company A should focus on the construction of a financial services center, so as to share finance as a back-end accounting platform in the accounting system to centralize data processing, collection, and the exchange of business in different regions. At the same time, we must develop an intelligent accounting decision support system and apply it in accounting practice. Secondly, we suggest to improve the level of human resources security. On the basis of the established accounting informatization professional job positions and supporting human resources, policies for accounting informatization talents should be formulated that are suitable for the long-term development of enterprises and that focus on cultivating and attracting informatized talents. Furthermore, we will improve the accounting informatization management system. Company A should improve its accounting information management system, including the management responsibilities of the accounting informatization department, hardware and software management, operation management, file management, anti-virus management, accounting data backup and security assurance, internal control management records, supervision, inspection, and assessment. In the process of accounting information management, Company A should follow the internal control management requirements, do a good job of the relevant control records, and put in place a supervision and inspection system.

## 6. Conclusions

In this paper, the approach to evaluating accounting informatization based on entropy in an intuitionistic fuzzy environment is proposed. Firstly, the evaluation index system of accounting informatization is constructed from the aspects of the informatization level in the accounting information progress as well as the accounting decision informatization. Considering the linguistic evaluation information, the intuitionistic fuzzy environment is used to model and deal with this input. Moreover, the weight of the evaluators is determined by intuitionistic fuzzy cross entropy, which reduces the personal bias. The application shows the feasibility and practicality of the proposed approach. Moreover, the weight of the evaluation index in the application can also be used in other companies and the suggestions made can also be used as valuable references. In future research, this approach can be used in more companies from different areas to make further improvements. Moreover, a way to determine the initial weight of experts more objectively should be developed.

## Figures and Tables

**Table 1:** Accounting information evaluation system.

First Level	Second Level	Third Level
Strategic position B1	Recognition and participation C1	High-level leaders’ importance D1 [25]
		The proficiency of the leadership in the application of the accounting information system D2 [26]
	Accounting informatization budget and planning C2	The construction of accounting informatization and the suitability of the operating environment D3 [26]
		Accounting informatization budget and actual completion D4 [27]
		Accounting informatization planning and actual completion D5 [27]
	Strategy and investment structure C3	Investment in accounting informatization D6 [28]
		Investment structure of accounting informatization D7 [29,30]
Infrastructure construction B2	Technical route of system architecture C4	The unification of the accounting information system and the overall system architecture technology roadmap D8 [25,31]
		Extensibility of the accounting information system architecture D9 [31]
	Level of infrastructure construction C5	Data integration of the accounting information system D10 [25]
		Construction of a financial sharing service platform D11 [32,33]
Accounting informatization implementation B3	Informatization level of the process of accounting information movement C6	Informatization of the accounting information generation process D12 [32]
		Informatization of the accounting information audit process D13 [34,35]
		Informatization of the accounting information exchange process D14 [36,37]
		Informatization of the accounting information mining process D15 [38]
		Informatization of the accounting information use process D16 [38]
	Accounting decision informatization C7	Accounting strategy decision informatization D17 [39]
		Accounting tactical decision informatization D18 [39]
		Accounting business decision informatization D19 [39]
Informatization guarantee B4	Safety and reliability C8	Construction of operation and maintenance management system D20 [40]
		Informatization security management system D21 [13,29,41,42]
		Safety management of accounting informatization personnel D22 [41]
		The degree of the application of new technology for accounting informatization D23 [43,44]
	Human resources C9	The penetration of accounting informatization technology D24 [45]
		Coverage of accounting informatization training D25 [46]
		The personnel policy of accounting informatization D26 [47,48]
Application benefits B5	Economic benefits C10	Direct economic benefits D27 [49,50]
		Indirect economic benefits D28 [51,52]
	Management benefits C11	Financial management informatization D29 [53]
		Internal control process informatization D30 [26]
	Social benefits C12	Save resources D31 [25]
		Social progress D32 [13]

**Table 2:** Conversion between linguistic variables and intuitionistic fuzzy numbers.

Language Evaluation Terms	Intuitionistic Fuzzy Numbers
Very Important/Very High	(0.90, 0.05)
Important/High	(0.75, 0.20)
Medium Important/Medium	(0.50, 0.40)
Unimportant/Low	(0.25, 0.60)
Very Unimportant/Very Low	(0.10, 0.80)

**Table 3:** Third-level evaluator weights.

Evaluators	The Evaluator Weights of Weight of Index	The Evaluator Weights of Evaluation Value	Evaluators	The Evaluator Weights of Weight of Index	The Evaluator Weights of Evaluation Value
Evaluator 1	0.0250	0.0382	Evaluator 16	0.0057	0.0167
Evaluator 2	0.0205	0.0315	Evaluator 17	0.0430	0.0407
Evaluator 3	0.0235	0.0367	Evaluator 18	0.0066	0.0114
Evaluator 4	0.0138	0.0091	Evaluator 19	0.0185	0.0276
Evaluator 5	0.0470	0.0253	Evaluator 20	0.0069	0.0173
Evaluator 6	0.0453	0.0276	Evaluator 21	0.0225	0.0345
Evaluator 7	0.0463	0.0268	Evaluator 22	0.0454	0.0290
Evaluator 8	0.0354	0.0551	Evaluator 23	0.0418	0.0438
Evaluator 9	0.0298	0.0499	Evaluator 24	0.0468	0.0254
Evaluator 10	0.0422	0.0413	Evaluator 25	0.0434	0.0368
Evaluator 11	0.0412	0.0442	Evaluator 26	0.0437	0.0352
Evaluator 12	0.0485	0.0204	Evaluator 27	0.0433	0.0363
Evaluator 13	0.0277	0.0424	Evaluator 28	0.0303	0.0492
Evaluator 14	0.0258	0.0383	Evaluator 29	0.0444	0.0333
Evaluator 15	0.0439	0.0338	Evaluator 30	0.0418	0.0421

**Table 4:** Weighted value of the third-level index.

Third-Level Evaluation Index	Evaluator Weighted Importance	Evaluator Weighted Evaluation	Comprehensive Evaluation Value	Score Function
High-level leaders’ importance D_1_	(0.7641, 0.1744)	(0.5773, 0.358)	(0.4412, 0.4700)	0.0155
The proficiency of the leadership in the application of the accounting information system D_2_	(0.6510, 0.2741)	(0.5249, 0.4058)	(0.3417, 0.5687)	−0.1822
The construction of accounting informatization and the suitability of the operating environment D_3_	(0.8052, 0.1451)	(0.5662, 0.3787)	(0.4559, 0.4688)	0.0247
Accounting informatization budget and actual completion D_4_	(0.7285, 0.2061)	(0.5714, 0.36)	(0.4162, 0.4919)	−0.0297
Accounting informatization planning and actual completion D_5_	(0.7362, 0.2108)	(0.5877, 0.3473)	(0.4327, 0.4849)	−0.0109
Investment in accounting informatization D_6_	(0.754, 0.2115)	(0.5116, 0.4148)	(0.3858, 0.5386)	−0.1150
Investment structure of accounting informatization D_7_	(0.711, 0.2451)	(0.3971, 0.5226)	(0.2823, 0.6396)	−0.3182
The unification of the accounting information system and the overall system architecture technology roadmap D_8_	(0.7956, 0.156)	(0.525, 0.4068)	(0.4177, 0.4994)	−0.0402
Extensibility of the accounting information system architecture D_9_	(0.7317, 0.2118)	(0.5408, 0.3991)	(0.3958, 0.5264)	−0.0917
Data integration of the accounting information system D_10_	(0.7929, 0.1614)	(0.5166, 0.4263)	(0.4096, 0.5189)	−0.0736
Construction of a financial sharing service platform D_11_	(0.7803, 0.1767)	(0.4819, 0.4512)	(0.376, 0.5482)	−0.1343
Informatization of accounting information generation process D_12_	(0.7423, 0.2099)	(0.4452, 0.4710)	(0.3305, 0.582)	−0.2078
Informatization of accounting information audit process D_13_	(0.7824, 0.1737)	(0.3980, 0.4979)	(0.3114, 0.5851)	−0.2219
Informatization of accounting information exchange process D_14_	(0.7761, 0.1760)	(0.4558, 0.4604)	(0.3538, 0.5553)	−0.1561
Informatization of accounting information mining process D_15_	(0.7654, 0.1877)	(0.394, 0.5182)	(0.3015, 0.6086)	−0.2621
Informatization of accounting information use process D_16_	(0.7522, 0.205)	(0.422, 0.4987)	(0.3175, 0.6015)	−0.2436
Accounting strategy decision informatization D_17_	(0.7685, 0.1884)	(0.3826, 0.5296)	(0.294, 0.6182)	−0.2803
Accounting tactical decision informatization D_18_	(0.737, 0.2182)	(0.4182, 0.5009)	(0.3082, 0.6098)	−0.2606
Accounting business decision informatization D_19_	(0.7314, 0.2236)	(0.4592, 0.4635)	(0.3358, 0.5835)	−0.2073
Construction of operation and maintenance management system D_20_	(0.7712, 0.1816)	(0.5627, 0.3531)	(0.434, 0.4706)	0.0111
Informatization security management system D_21_	(0.7621, 0.1916)	(0.6339, 0.2923)	(0.4831, 0.4279)	0.0997
Safety management of accounting informatization personnel D_22_	(0.7897, 0.1729)	(0.489, 0.431)	(0.3862, 0.5294)	−0.1010
The application degree of the new technology of accounting informatization D_23_	(0.6809, 0.2656)	(0.4619, 0.4634)	(0.3145, 0.6059)	−0.2516
The penetration of accounting informatization technology D_24_	(0.6978, 0.2434)	(0.4735, 0.4412)	(0.3304, 0.5772)	−0.2006
Coverage of accounting informatization training D_25_	(0.6547, 0.2846)	(0.4192, 0.5089)	(0.2745, 0.6487)	−0.3358
The policy of accounting informatization personnel D_26_	(0.6434, 0.3024)	(0.4805, 0.4555)	(0.3092, 0.6201)	−0.2756
Direct economic benefits D_27_	(0.7515, 0.1824)	(0.4662, 0.4753)	(0.3503, 0.571)	−0.1813
Indirect economic benefits D_28_	(0.7662, 0.183)	(0.4917, 0.4525)	(0.3768, 0.5527)	−0.1406
Financial management informatization D_29_	(0.794, 0.1609)	(0.5147, 0.425)	(0.4086, 0.5175)	−0.0720
Internal control process informatization D_30_	(0.7544, 0.2023)	(0.5762, 0.3707)	(0.4347, 0.498)	−0.0296
Save resources D_31_	(0.5608, 0.4096)	(0.434, 0.4919)	(0.2434, 0.7)	−0.4283
Social progress D_32_	(0.561, 0.3894)	(0.3679, 0.5578)	(0.2064, 0.73)	−0.4918
